# Genetic investigations on 8 patients affected by ring 20 chromosome syndrome

**DOI:** 10.1186/1471-2350-11-146

**Published:** 2010-10-12

**Authors:** Daniela Giardino, Aglaia Vignoli, Lucia Ballarati, Maria Paola Recalcati, Silvia Russo, Nicole Camporeale, Margherita Marchi, Palma Finelli, Patrizia Accorsi, Lucio Giordano, Francesca La Briola, Valentina Chiesa, Maria Paola Canevini, Lidia Larizza

**Affiliations:** 1 Laboratorio di Citogenetica Medica e Genetica Molecolare, IRCCS Istituto Auxologico Italiano Milan, Italy; 2 U.O. Neurologia 2, A.O. San Paolo - DMCO, Università degli Studi di Milano, Milan, Italy; 3 Dipartimento di Biologia e Genetica per le Scienze Mediche, Università degli Studi di Milano, Milan, Italy; 4 Unità Operativa di Neuropsichiatria dell'Infanzia e dell'Adolescenza, Spedali Civili, Brescia, Italy; 5 Dip. Medicina Chirurgia e Odontoiatria, A.O. San Paolo, Università degli Studi di Milano, Milan, Italy

## Abstract

**Background:**

Mosaic Chromosome 20 ring [r(20)] is a chromosomal disorder associated with a rare syndrome characterized by a typical seizure phenotype, a particular electroclinical pattern, cognitive impairment, behavioural problems and absence of a consistent pattern of dysmorphology. The pathogenic mechanism underlying seizures disorders in r(20) syndrome is still unknown. We performed a detailed clinical and genetic study on 8 patients with r(20) chromosome, aimed at detecting the genetic mechanism underlying r(20) syndrome.

**Methods:**

We submitted 8 subjects with a previous diagnosis of ring 20 chromosome mosaicism to a clinical re-evaluation, followed by cytogenetic, FISH, array-CGH and molecular analyses. The genetic study was also extended to their available parents.

**Results:**

FISH and array-CGH experiments indicate that cryptic deletions on chromosome 20 are not the cause of the r(20) chromosome associated disease. Moreover, no evidence of chromosome 20 uniparental disomy was found. Analysis of FISH signals given by variant in size alphoid tandem repeats probes on the normal chromosome 20 and the r(20) chromosome in the mosaic carriers suggests that the r(20) chromosome is the same chromosome not circularized in the "normal" cell line.

**Conclusions:**

Higher percentages of r(20) chromosome cells were observed to be related with precocious age at seizure onset and with resistance to antiepileptic drug treatment. Behavioural problems also seem to be associated with higher percentages of r(20) chromosome cells. Our results suggest that an epigenetic mechanism perturbing the expression of genes close to the telomeric regions, rather than deletion of genes located at the distal 20p and/or 20q regions, may underlie the manifestation of r(20) syndrome.

## Background

Mosaic Chromosome 20 ring [r(20)] is a chromosomal disorder that has been associated with a rare syndrome characterized by a typical seizure phenotype consisting of complex partial seizures, a particular electroclinical pattern, cognitive impairment, behavioural problems and absence of a consistent pattern of dysmorphology [1-4]. Over 60 cases have been reported up to now, mostly sporadic, with mosaicism for a de novo r(20) chromosome resulting in refractory epilepsy with non convulsive status epilepticus (NCSE) and cognitive problems [5-7]. A reassessment of the published cases with [r(20)] syndrome indicates that the development is generally normal or mildly delayed but it is followed by cognitive and behavioural decline after seizure onset indicating that the clinical disorder could be interpreted as an epileptic encephalopathy [8].

At the chromosomal level, r(20) chromosome replaces one of the two chromosomes 20 in a percentage of cells, ranging from 1 to 100% in lymphocytes. The relation between the variable mosaicism and the clinical phenotype has been and is still controversial [6,9,10].

The pathogenic mechanism underlying the seizures disorder in r(20) syndrome is unknown. Only a few cases have been investigated with FISH technique oriented at detecting subtelomeric deletions of chromosome 20 associated with ring formation and/or deletion of the 20qter epilepsy genes CHRNA4 and KCNQ2 [4,11-14]. Here we describe the clinical presentation of 8 patients, 6 sporadic cases and two family members found to be carriers of r(20) chromosome mosaicism. We report on the conventional cytogenetics, FISH characterization and genotyping of chromosome 20 polymorphic markers of probands and parents aimed at detecting the genetic mechanism underlying r(20) syndrome. Array CGH, which has not yet been applied to patients carrying r(20) chromosome was also used to interrogate the overall genome of the patients and rule out chromosome 20 copy number imbalances.

## Methods

### Patient recruitment

The study was approved by the Ethics Committee of Istituto Auxologico Italiano (RC 08C703_2007), participating subjects gave informed consent and procedures followed were in accordance with institutional guidelines. Eight probands were recruited for the genetic analyses by the two neurological units involved in this study. An informed consent was also obtained from parents and relatives submitted to genetic tests.

### Cognitive assessments

Cognitive assessment of patients was performed using Bayley Scales of Infant Development and WISC-R Intelligence Scale for pediatric age or Wais Intelligence Scale for Adult Age [15-17].

### Video-EEG recordings

Patients underwent EEG video-polygraphic recordings during wakefulness and sleep using a computerized EEG System. Scalp electrodes were positioned according to the international 10/20 system plus EMG electrodes for deltoid muscles and/or distal muscles, electrocardiogram and breathing effort.

We performed several video-EEG (VEEG) monitoring and sleep recordings in each patient.

### Magnetic Resonance Imaging (MRI) - Brain

Brain MRIs were obtained in FL, DG, CD, BV, PE and BE patients. Because the imaging studies were performed at different institutions, the imaging parameters varied slightly. However, the patients underwent brain MRI with 0.5-tesla or 1.5-tesla apparatus. Noncontrast-enhanced sagittal, axial and coronal T1-weighted images, and axial T2-weighted images with 4-6 mm section thickness were available.

### Cytogenetic, FISH and array-CGH analyses

The cytogenetic analysis was performed using Q-banding on lymphocytes and fibroblasts from each proband and parents, when available. Conventional protocols were used to set up the cultures and chromosome preparations. Both commercial and homemade probes were used for the FISH analyses. The commercial probes were pantelomeric and chromosome 20-specific α-satellite DNA and subtelomeric probes (Vysis, Chicago, IL). The FISH experiments were carried out according to the suppliers' protocols. FISH to detect the integrity of the CHRNA4 and KCNQ2 regions was performed with RCP11-939M14 and RCP11-358D14 BAC clones selected according to the UCSC Genome Browser, March 2006 release, and provided by the University of Bari, Italy (Resources for Molecular Cytogenetics: http://www.biologia.uniba.it/rmc/). The order of probes from cen to tel is RCP11-939M14, RCP11-358D14 and 20q subtelomeric probe (Vysis, Chicago, IL). The distance from RCP11-939M14 to RCP11-358D14 is ~20 kb and ~500 kb from RCP11-358D14 to the 20q subtelomeric commercial probe. BAC FISH experiments were executed according to Lichter et al. with minor modifications[18]. Array-CGH was performed on genomic DNA from probands, extracted from peripheral blood mononucleate cells with the GenElute™ Blood Genomic DNA kit (Sigma-Aldrich, St Louis, Missouri, USA) according to the instructions of the supplier. The Human Genome CGH Microarray Kit 244A was employed according to the protocol provided by Agilent (Agilent technologies, Santa Clara, CA). This platform is a high-resolution 60-mer oligonucleotide-based microarray allowing genome-wide survey and molecular profiling of genomic imbalances with ~30 Kb average resolution. For chromosome 20p and 20q subtelomeric regions the average resolution is ~30 kb and ~50 kb respectively, with the most distal oligonucleotide on 20p and 20q located respectively at ~9 kb and ~57 kb from the chromosome ends. The arrays were analyzed with the Agilent Scanner Control (v 7.0) and the Feature Extraction software (v 9.5.3.1). Graphical overview was obtained using the DNA Analytics software (v4.0). The unbalanced regions pointed out by the analysis were studied by means of the Database of Genomic Variants http://projects.tcag.ca/variation and the UCSC Genome Browser http://genome.ucsc.edu/, March 2006 release.

### Molecular analysis

DNA was extracted from peripheral blood leukocytes of the probands and parents by using automated system Tecan Freedom Evo (Mannedorf, Swizerland), GeneCatcher(tm) gDNA 96 × 10 ml Automated Blood Kit, Invitrogen (Carlsbad, CA). A first panel of fluorescinated primers, D20S117, D20S889, D20S115, D20S107, D20S119, D20S178, D20S196, D20S100, and D20S173, selected from the ABI Prism Linkage Mapping Set and spanning the chromosomal regions 20p12.3-p13 and 20q12-q13.33 at a minimum distance of 10 cM, was investigated in each trio of parents and proband. In the familial cases (FL and FMA) and in MM, markers D20S186, D20S112, D20S195 D20S171 were added in order to replace the uninformative ones. PCRs were performed according to standard protocols for the microsatellite panel advised by Aplera (ABI PRISM(r) Linkage Mapping Set version 2.5 Panel). PCR products were separated by capillary electrophoresis on the Automated ABI 310 Sequencer (PE Applied Bio-system, Foster City, CA, USA).

## Results

### Clinical data

The clinical characteristics and the r(20) chromosome percentages observed in lymphocytes and fibroblasts of the enrolled patients are summarized in Table 1. Eight patients (2 males, 6 females, mean age 24 years (range 3-59 years), were included in the study. A detailed clinical description of patients FL, FMA and BD was provided by Canevini et al, as cases 1, 2, 3 respectively, while that of CD and DG has recently been published as cases 2 and 3 [4,8]. Only MM patient showed facial dysmorphisms with frontal bossing, inner epicanthal folds and a low nasal bridge.

**Table 1:** Clinical phenotypes of the patients enrolled for the study and conventional cytogenetics results

Case	Sex	r(20) %^a^	r(20) %^b^	Age at study (year)	Age at diagnosis (year)	IQ	Growth retardation	Facial dysmorphisms	Major Malform-ations	Behaviour problem	Seizures	Onset age (year)	Type of epilepsy	Clinical course	NCSE	Seizures frequency	EEG
**FL°**	M	71	81	30	17	60	-	-	-	+	+	5	Focal Epilepsy	Drug-resistance	+	Monthly	Fronto-temporal sharp-waves
DG*	F	67	58	22	13	69	-	-	-	+	+	4	Focal Epilepsy	Drug-resistance	+	Weekly	Frontal sharp-waves
CD*	F	42	46	13	10	80	-	-	-	+	+	9	Focal Epilepsy	Drug-resistance	+	Daily	Frontal sharp-waves
**BV**	F	42	37	19	12	71	+	-	+	+**	+	12	Focal epilepsy	Drug-resistance	+	Daily	Frontal sharp-waves
**PE**	F	34	5	16	14	80	-	-	-	+	+	10	Focal epilepsy	Drug-resistance	+	Weekly	Frontal sharp-waves
**MM**	F	30	NE	3	PD	101	+	+	-	-	-	-	-	-	-	-	Normal
**FMA°**	F	9	0	59	44	83	-	-	-	-	+	11	Focal epilepsy	Controlled	-	-	Fronto-temporal sharp-waves
**BD°**	M	8	3	34	21	N	-	-	-	-	+	17	ND	Controlled	-	-	Fronto-temporal sharp-waves

^a ^% observed in proband's lymphocytes; ^b ^% observed in proband's fibroblasts; ° patients described in: Canevini MP et al. [4]; * patients described in: Vignoli A et al [8]; N Normal; ** Behavioural problems started at age 7 before seizure onset; ND: not determined; NE Not evaluated (prenatal diagnosis): 60% of cells with r(20) on chorionic villi and 14% on amniocytes.; NCSE Non Convulsive Status Epilepticus; PD Prenatal diagnosis of r(20) mosaicism.

A structural abnormality of the internal ear associated with deafness has been observed in BV. Growth retardation has been detected in BV and MM. FMA and BD show a normal IQ, while in the remaining cases IQ is below normal or in the lower range (see Table 1). Epilepsy was the symptom that led to the evaluation of all patients except for the youngest child, MM, aged 3 years, who has been followed-up after a r(20) mosaicism prenatal diagnosis and is not yet epileptic. The average age at seizure onset was 9 years (range 4-17 years). The majority of the patients experienced more than one seizure type, including complex partial seizures, atypical absences, generalized tonic-clonic seizures and focal motor seizures. Five patients (FL, DG, CD, BV, PE) showed NCSE in the course of their disease. Video-EEG recordings demonstrated frontal and/or fronto-temporal abnormalities in all patients with epilepsy. Seizures and/or NCSE have been recorded in 5 patients (FL, DG, CD, BV, PE).

In 5 out of 7 patients, the epilepsy was drug resistant, with very frequent seizures in most cases (Table 1). After seizure onset, all patients except FMA and BD developed deterioration of cognitive abilities with loss of previously acquired skills. None of the patients showed progressive motor impairment. Five patients presented with behavioural problems: autistic features with loss of social skills and impaired interpersonal relationships were observed in FL, CD, BV. Attention deficit was evident in FL, CD and DG, irritability in BE and depressive traits in BV. Brain magnetic resonance was performed in FL, DG, CD, BV, PE and BE with normal results. FMA underwent a brain CT scan that provided normal result. Excluding the familiar case FL, all parents were reported to be healthy.

### Cytogenetic and FISH analyses

Table 1 lists the results of the conventional cytogenetic analyses performed on our 8 patients. A *de novo *origin of r(20) chromosome could be established in four cases: CD, DG, BD and MM by the analyses of no less than 60 metaphase lymphocytes from their parents. Conversely FL inherited the r(20) chromosome from his mother (FMA): the maternal grandmother was excluded as being a carrier by scoring 72 lymphocyte metaphases, while this investigation was precluded on the deceased maternal grandfather. The parents of the two remaining patients, PE and BV, were unavailable for the cytogenetic study.

The percentage of cells with r(20) chromosome, detected in the probands by scanning no less than 60 metaphases from peripheral blood and 30 metaphases from skin biopsy, was found to range from 8 to 71% in lymphocytes and from 0 to 81% in fibroblasts. Patients FL and BD showed respectively the highest and lowest percentages in the analyzed tissues. The percentage of cells with r(20) chromosome was similar in peripheral blood and fibroblasts of CD and BV, while PE showed the widest variation. The percentage of r(20) chromosome cells appears to be dynamic across time as FL, FMA and DB show on lymphocytes 71, 9 and 8% of r(20) chromosome cells as compared to 83, 12 and 16% which were reported eleven years before by Canevini et al. [4], 53% of r(20) chromosome was detected on peripheral blood at diagnosis in CD and 26% in DG as compared with 42% and 67% respectively observed in the present study (Table 1).

**Figure 1:**
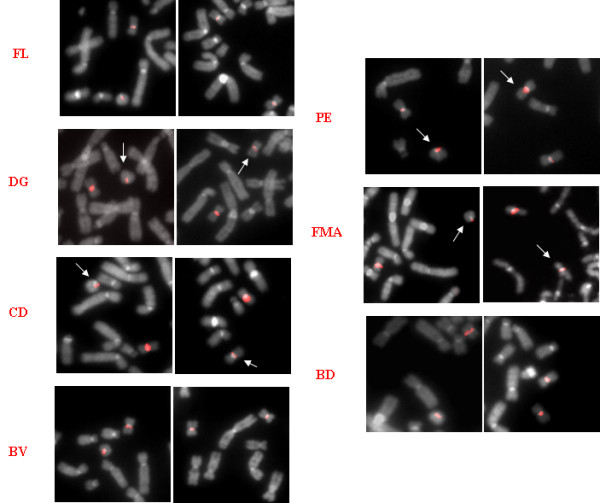
**FISH analysis with chromosome 20-specific α-satellite DNA probes**. FISH results on r(20), indicating alphoid-specific heteromorphism suitable to discriminate between the two homologous chromosomes 20 (arrowed) in DG, CD, PE and FMA patients. In FL, BV and BD no polymorphic signals on chromosome 20 homologous have been identified. See text for explanation

FISH investigations with a chromosome 20-specific α-satellite DNA probes showed the derivation of the ring chromosomes from chromosome 20 (Figure 1). The analysis of at least 100 interphase nuclei and 60 lymphocyte metaphases of each patient indicated that no more than 1% of cells were monosomic for chromosome 20 due to the loss of r(20) chromosome because of its mitotic instability. The presence of variant in size alphoid tandem repeats monitored by FISH signals of different intensities (low/high) was also observed on chromosome 20 centromeres (Figure 1). An alphoid-specific heteromorphism suitable to discriminate between the two homologous chromosomes 20 was detected in DG, CD, PE and FMA patients, whereas in FL and BV the signals were similar in size on both chromosome 20 centromeres (Figure 1). Careful scoring of about 15 metaphases from the informative patients allowed us to distinguish the apparently normal chromosome 20 from the r(20) chromosome using the "identity" of the structure of the centromeric constriction. Indeed the "low" intensity signal discriminating r(20) chromosome from its "high" signal normal homologous was present in the not "circularized" chromosome 20 in the cell line with normal karyotype of DG, CD and FMA likewise the "high" intensity signal of r(20) chromosome of PE (Figure 1).

**Figure 2:**
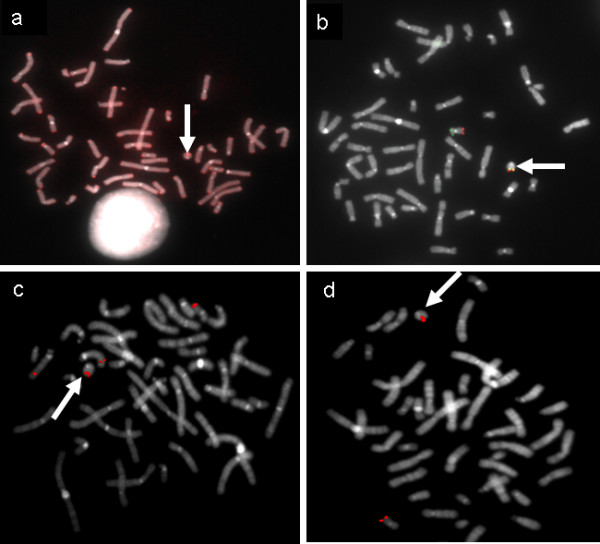
**Example of the FISH results obtained for each proband on ring 20 chromosome**. **A**) pan-telomeric probe; **B**) 20p (green signal) and 20q (red signal) subtelomeric specific probes; **C**) RCP11-939M14 and **D**) RCP11-358D14 BAC clones targeting CHRNA4 and KCNQ2 epilepsy genes respectively. The presence of the signals on r(20) (arrowed) demonstrates the integrity of the investigated regions. In panel **C**, RCP11-939M14 clone cross-hybridizes with chromosome 15.

FISH with pan-telomeric and subtelomeric specific probes was performed on each patient with similar results: the presence of signals on r(20) chromosome demonstrated the integrity of the target regions in the ring chromosome (Figure 2a and 2b) as well as in both chromosomes 20 of the karyotypically normal cell line. In addition the finding of gene-specific RCP11-939M14 and RCP11-358D14 BAC signals on the rearranged chromosome as well as on both chromosomes 20 of the karyotypically normal cell line indicated that CHRNA4 and KCNQ2 gene sequences are maintained in both cell lines (Figure 2c and 2d).

### Array-CGH analysis

**Figure 3:**
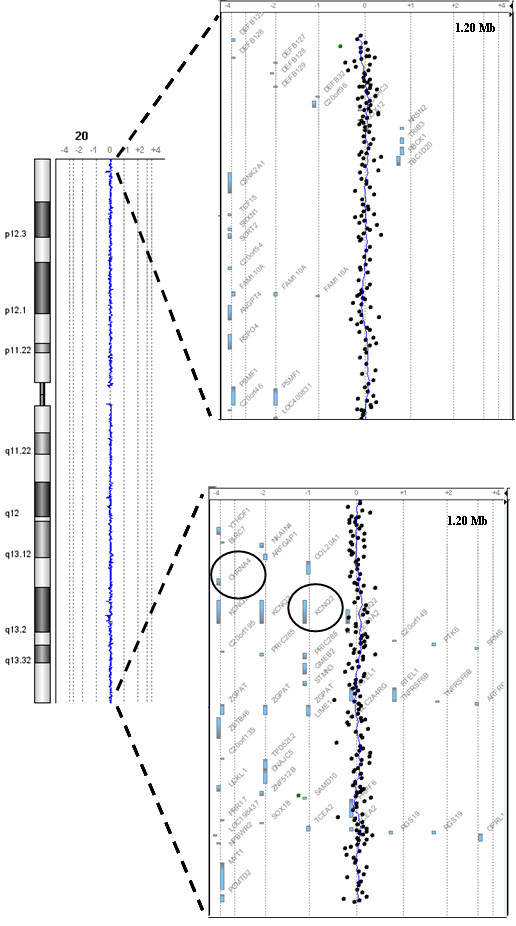
**Array-CGH chromosome 20 profile**. Example of normal profile observed in all patients and magnifications of the 1,2 Mb distal 20p and 20q regions. CHRNA4 and KCNQ2 genes on 20q are circled.

**Figure 4:**
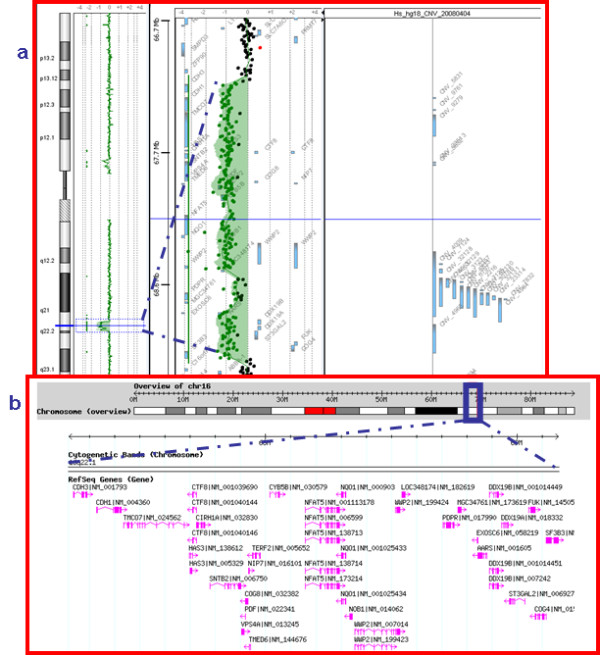
**Array-CGH chromosome 16 profile**. **A**) Genomic imbalances on patient BV chromosome 16, with magnification of the deleted region and **B**) the contained genes.

Array-CGH analyses revealed a chromosome 20 normal profile in all patients (Figure 3). Surprisingly, BV was found to be carrier of a 1.9 Mb deletion between 67228076 and 69164712 nucleotides (hg18 NCBI build36) on chromosome 16q22.1 (Figure 4a and 4b). The 1.9 Mb deleted region identified in BV contains 42 genes (Figure 4b), 27 of which with a known function, making it hard to link deletion for the genes with the patient's phenotype. Since the proband's mother had died, assessment of the *de novo *origin of the anomaly could not be established.

### Molecular analysis

**Figure 5:**
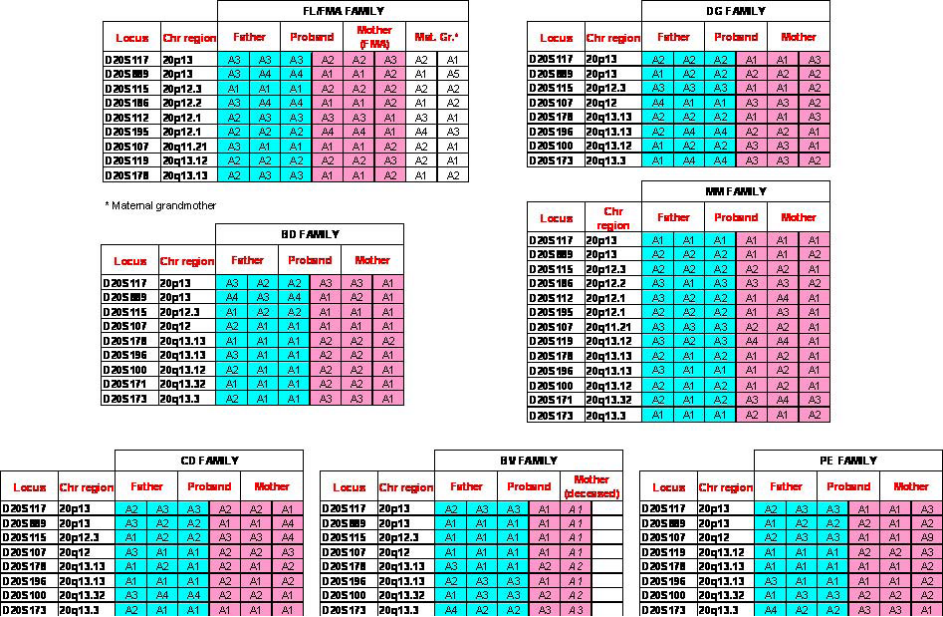
**Results of segregation analysis from parents to probands of chromosome 20 microsatellites**.

Occurrence of either whole or segmental chromosome 20 uniparental disomy (UPD) was investigated by segregation analysis of polymorphic loci from parents to proband. In all cases the informative markers evidenced a biparental contribution. In BV family microsatellite analysis only allowed exclusion of paternal UPD, because the mother was deceased. Segregation analysis from FL grandmother to FL through FMA indicated the biparental contribution of chromosome 20 and the absence of recombination along the chromosome, which appears to be transmitted in an identical form across two generations (Figure 5).

## Discussion and Conclusion

In the present study we have characterized on the genetic aspects and clinical ground the largest cohort of ring 20 patients reported till now. All patients but the youngest one (MM, 3 years old) presented the typical seizure disorder associated with r(20) chromosome, with a mean age at seizure onset of 9 years. Epilepsy onset has been reported in paediatric, adolescent and adult age. Patients with an early epilepsy onset (within the first year of age) have been reviewed by Ville et al: all exhibited severe psychomotor delay before seizure onset and a percentage of r(20) chromosome cells ranging from 87 to 100% [19].

More than 60 cases of patients with a non supernumerary r(20) chromosome mosaicism have been reported, but the great majority have been investigated only with conventional cytogenetic analysis.

Ring(20) syndrome is characterized by phenotypic variability, particularly concerning dysmorphism, malformations, mental retardation and behavioural disturbances [20]. The severity of clinical features has been ascribed to high percentages of r(20) chromosome abnormal cells, ring instability and presence or absence of chromosome 20 deleted regions [10,13,21]. Previously reported patients showed a percentage of r(20) chromosome cells ranging from 1 to 100% in peripheral blood. Generally, cases with 100% anomalous cells exhibited more severe development and psychomotor delay, starting from the first months of life, before seizure onset[19]. Previous studies failed to reach a consensus regarding the relationship between the percentage of mosaicism and the manifested phenotype. No correlation was found between IQ and age at seizure onset in two independent reports,[6,9] whereas Nishiwaki et al. [10] concluded that the mosaicism ratio is significantly associated with IQ, age at seizure onset and malformations, but not with the response to antiepileptic drug treatment. A positive correlation between age at seizure onset and rate of r(20) mosaicism was also observed by Herrgard et al. [11]. In our patient cohort the percentage of r(20) chromosome cells covered a wide range, from 8 to 71% in peripheral blood. In seven out of eight investigated cases the percentage of r(20) chromosome could be also computed on fibroblasts, making the level of mosaicism representative of two different cell lineages. Our results indicate that higher percentages of r(20) chromosome cells are observed in probands with younger age at seizure onset and seem also to correlate with resistance to antiepileptic drug treatment, agreeing with the findings of Nishiwaki et al. and Herrgard et al. [10,11]. The two patients from our series showing a lower degree of r(20) mosaicism developed seizures at an older age, epilepsy was controlled and they are the only two patients without mental impairment in adult age. These cases further demonstrate the fundamental role of seizures in determining cognitive impairment in r(20) syndrome [8]. Behavioural problems also appear in our patients to be related with higher percentages of r(20) chromosome cells. A major malformation was observed only in BV, who is the patient with a concurrent 1.9 Mb deletion on 16q chromosome (Figure 4a and 4b), confirming that this finding is likely to be coincidental and not characteristic of the r(20) mosaic patients in addition to the dysmorphism, which were observed just in BV and MM.

It has been suggested that the presence of chromosome 20 monosomic cells generated by r(20) chromosome instability during mitosis might be implicated in the phenotypic manifestations of r(20) syndrome [13] as well as in the so call "ring syndrome" [22]. However the FISH results on our series revealed only about 1% of monosomic cells in each patient, different to the significantly higher percentage (8%) detected by Elghezal et al. [13] but in agreement with the recently reported conclusions weakening the hypothesis of the "ring syndrome" phenotype caused by ring instability [23]. Ring (20) chromosome mosaicism generally represents a *de novo *event. To date, only three families with several carrier members have been published. A clinically unaffected carrier mother transmitting the r(20) chromosome to two siblings has been reported by Back et al. [24], while in the familial cases described by Herrgard et al. [11] the two siblings inherited the r(20) chromosome from their affected mother. Canevini et al. reported a son (FM in this study) inheriting the r(20) chromosome from his less severely affected mother (FMA in this study) [4]. The concomitance of a cell line with two apparently "normal" chromosomes 20 in patients with an inherited r(20) chromosome is still an open question. A possible explanation is that the "normal" cell line is isodisomic for chromosome 20. This condition may have occurred by a rescue mechanism rectifying through duplication of the normal chromosome 20 the monosomic cell originated by loss of r(20) chromosome. Another mechanism leading to a mosaicism with a normal cell line in r(20) chromosome inherited cases is the trisomy rescue in a 47,+r(20) conceptus consequent to a random loss of r(20) chromosome or normal chromosome 20 in different cells during mitosis in early development. We have thus searched for putative UPD20 in our mosaic familial and de novo cases by segregation analysis of chromosome 20 microsatellites from parents to probands. To the best of our knowledge, this is the first study in which UPD20 has been evaluated in patients affected by r(20) disease. Our results do not support the UPD20 hypothesis, although they suggest through the haplotype signature that the r(20) chromosome is the same chromosome that appears and is not circularized in the "normal" cell line (Figure 5). This hypothesis is not contradicted by the FISH results obtained using a 20-specific alphoid probe able to visualize different signal intensities when the two centromeric constrictions are built up with a different number of alphoid repeats (Figure 1). This finding might have the following implications: a) r(20) patients have two cell lines differing only on a morphological but not a genetic point of view; b) one of the two chromosomes 20 has a genetic motif, yet unknown, inducing both the "circularization" and the "re-opening". The direct physical link between distal 20p and 20q segments in the ring structure may disturb the correct expression or the regulation of genes located nearly the telomeric regions. It may also prime the phenotypic manifestation of the syndrome. The mosaic condition of the patient inheriting the r(20) chromosome from his mother may evidence the plasticity of the circularized chromosome.

To date only 10 r(20) patients, including 3 familial cases [11], have been investigated by FISH techniques [4,11-13,21,25] and one with telomere PRINS (primer in situ DNA synthesis) [26]. Three out of 7 cases were found to carry a r(20) chromosome with deletion of the telomeric regions [13,21,26]. In 9 probands, the subtelomeric 20p and 20q regions have been studied and the r(20) chromosome has been shown to maintain these sequences in all carriers [4,11,13,14] but one [25].

Ring(20) chromosome without deletion of the CHRNA4 and KCNQ2 epilepsy genes was found in the only two patients so far investigated for this aspect [12,13]. In our series of patients FISH analyses indicated no deletions of the telomeric and subtelomeric chromosome 20 regions or deletion of CHRNA4 and KCNQ2 genes. However, small deletions in their promoters or in regulatory regions can't be excluded. No evidence for loss of chromosome 20 genetic material was also provided by the array-CGH analysis, even if the possibility of a subtle submicroscopic deletion on r(20) chromosome can't be ruled out, since array-CGH may fail to detected low level r(20) cell mosaicism.

Our wide genetic study includes UPD20 evaluation and array-CGH analysis which had never been performed before on r(20) patients. We speculate that the mechanism/mechanisms underlying the seizure disorder in r(20) carriers must be related on the conversion of ring 20 to normal chromosome 20 morphology and vice versa. This speculation should be corroborated by studying further familial cases. Moreover, the hypothesis that tandem repeats of telomere DNA forming a heterochromatic structure may silence subtelomeric CHRNA4 and KCNQ2 genes or their regulatory components should be investigated. The presence of other epileptic genes mapping to chromosome 20 also deserves to be considered in the future within the panel of players in the pathogenetic cascade responsible for r(20) syndrome manifestations.

## List of abbreviations used

BAC: Bacterial artificial chromosome; CGH: Comparative genomic hybridization; CHRNA4: Cholinergic receptor, nicotinic, alpha 4; EEG: Electroencephalography; EMG: Electromyography; FISH: Fluorescence in situ hybridization; KCNQ2: Potassium voltage-gated channel, KQT-like subfamily, member 2; MRI: Magnetic Resonance Imaging; NCSE: Non convulsive status epilepticus; PRINS: Primer in situ DNA synthesis; UPD: Uniparental disomy; VEEG: Video- Electroencephalography; WISC R.: Wechsler Intelligence Scale for Children Revised.

## Authors' statement

The authors declare that the initials used to identify patients do not correspond to the patients' actual names.

## Competing interests

The authors declare that they have no competing interests.

## Authors' contributions

DG participated in the conception, design and coordination of the study, in interpretation of data and drafted the manuscript; AV contributed with patients recruitment, clinical evaluation and helped to draft the manuscript; LB, MPR and PF carried out cytogenetic analyses on lymphocytes and fibroblasts of the patients and their parents and interpreted FISH and array results; SR coordinated the UPD study and helped to draft the manuscript; NC performed blood and fibroblast cultures and carried out FISH and array experiments; MM carried out microsatellite analyses of all the families; PA, LG, FLB and VC contributed with patients recruitment and carried out cognitive assessments, Video-EEG recordings and Magnetic Resonance Imaging; MPC contributed with clinical evaluation of the probands and participated in the revision of clinical data reported in the manuscript; LL participated in the conception, design and coordination of the study, in the revision of the manuscript and final approval of the version. All the authors read and approved the final manuscript.

## Pre-publication history

The pre-publication history for this paper can be accessed here:

http://www.biomedcentral.com/1471-2350/11/146/prepub
